# Sirolimus versus cyclosporine in haploidentical stem cell transplantation with posttransplant cyclophosphamide and mycophenolate mofetil as graft‐versus‐host disease prophylaxis

**DOI:** 10.1002/jha2.183

**Published:** 2021-03-18

**Authors:** Rafael Hernani, José Luis Piñana, Ariadna Pérez, Abdiel Quintero, Juan Montoro, Juan C. Hernández‐Boluda, Carlos Carretero, Aitana Balaguer‐Roselló, Manuel Guerreiro, Ignacio Lorenzo, Cristóbal Aguilar, Estela Giménez, David Navarro, Miguel A. Sanz, Jaime Sanz, Carlos Solano

**Affiliations:** ^1^ Department of Haematology Hospital Clínico Universitario Institute for Research INCLIVA Valencia Spain; ^2^ Department of Haematology Hospital Universitari i Politècnic La Fe Valencia Spain; ^3^ Microbiology Service Hospital Clínico Universitario Institute for Research INCLIVA Valencia Spain; ^4^ Department of Medicine University of Valencia Valencia Spain; ^5^ Department of Microbiology University of Valencia Valencia Spain; ^6^ CIBERONC Instituto Carlos III Madrid Spain

**Keywords:** cyclosporine, graft‐versus‐host disease prophylaxis, haploidentical transplantation, posttransplant cyclophosphamide, sirolimus, toxicity, viral infections

## Abstract

Sirolimus has emerged as an alternative to calcineurin inhibitors‐based (CNI) graft‐versus‐host disease (GVHD) prophylaxis. This retrospective study compares the outcome of 133 consecutive adult patients with haematological malignancies undergoing haploidentical stem cell transplantation with posttransplant cyclophosphamide (PTCy) and mycophenolate mofetil (MMF), combined with cyclosporine A (PTCy–CsA–MMF, *n* = 67) or sirolimus (PTCy–Sir–MMF, *n* = 66) as GVHD prophylaxis strategy. The median follow‐up was 48 (range 22–83) and 13 (range 3–33) months, respectively. PTCy–CsA–MMF was associated in multivariate analyses with a higher risk of acute kidney injury (HR 2.1, 95% CI, 1.21–3.57, *p* = .008) and thrombotic microangiopathy (HR 12.5, 95% CI, 1.66–93.5, *p* = .014), whereas PTCy–Sir–MMF was associated with a higher risk of hepatic sinusoidal obstruction syndrome (SOS) (HR 10.8, 95% CI, 1.52–77, *p* = .018), especially late‐onset forms, which totally resolved and none of the patients needed discontinuation of sirolimus. Two SOS‐related deaths were detected, both in the PTCy–CsA–MMF subgroup. Both GVHD prophylaxis strategies were otherwise comparable in terms of engraftment, GVHD incidence and survival.

## 1 INTRODUCTION

High‐dose posttransplant cyclophosphamide (PTCy) combined with calcineurin inhibitors (CNI), such as cyclosporine A (CsA) or tacrolimus, is a well‐established graft‐versus‐host disease (GVHD) prophylaxis in the setting of haploidentical stem cell transplantation (haplo‐SCT) [1, 2].

In recent years, sirolimus has been explored as an alternative to CsA‐based prophylaxis and some advantages have been suggested, including a more favourable toxicity profile, especially in terms of renal toxicity [3], better immune reconstitution [4, 5], more powerful graft‐versus‐lymphoma effect [6, 7], and reduced incidence of *Cytomegalovirus* (CMV) DNAemia [8].

Encouraging results have recently been reported using CNI‐free GVHD prophylaxis with PTCy and sirolimus in patients undergoing SCT from both HLA‐matched related and unrelated donors [7, 9], as well as haploidentical donors [10, 11, 12]. However, a formal comparison of CNI‐free with CNI‐based GVHD prophylaxis is still missing.

In the present study, we aimed to compare outcomes and toxicities of patients undergoing haplo‐SCT at two institutions using PTCy, sirolimus, and mycophenolate mofetil (PTCy–Sir–MMF) with a historical cohort of haplo‐SCT with PTCy, CsA and MMF (PTCy–CsA–MMF).

## 2 PATIENTS AND METHODS

### 2.1 Patients and eligibility criteria

In this retrospective study, we included two consecutive cohorts of recipients who underwent haplo‐SCT with PTCy–CsA–MMF or PTCy–Sir–MMF as GVHD prophylaxis in two transplant centres (Hospital Clínico Universitario [*n* = 59] and Hospital Universitari i Politècnic La Fe [*n* = 74], both in Valencia, Spain). The first cohort (PTCy–CsA–MMF) was allografted from October 2012 to December 2016 and the second (PTCy–Sir–MMF) cohort from January 2017 to December 2019. According to the Declaration of Helsinki, the Research Ethics Board of Hospital Clínico Universitario approved the study (reference code 2020/160).

Inclusion criteria were (i) age >15 years; (ii) haematological malignancy; (iii) Eastern Cooperative Oncology Group performance status ≤2 without uncontrolled infection; (iv) written informed consent.

Exclusion criteria were (i) previous allogeneic SCT; (ii) sequential chemotherapy and conditioning regimen for refractory acute myeloid leukaemia patients; (iii) pretransplant organ dysfunction >grade 2 as defined by National Cancer Institute (NCI) criteria (Common Toxicity Criteria version 5).

### 2.2 Donor selection and source of stem cells

Selection of a haploidentical donor did not vary between periods and it has previously been described in detail [12]. Briefly, a haploidentical family donor was selected according to the following considerations (in order of preference): absence of recipient HLA antibodies against donor antigens, male sex, younger age, matched CMV serostatus and matched ABO group. Initially, peripheral blood mobilised CD34+ stem cells were used at both transplant centres. In mid‐2019, bone marrow source was prioritised, when feasible, at both centres in elderly donors without comorbid conditions, in order to mitigate the development of severe GVHD.

**Key Points**

- Both prophylactic regimens provide comparable efficacy preventing GVHD.
- PTCy–CsA–MMF was associated with a higher risk of acute kidney injury and thrombotic microangiopathy.
- PTCy–Sir–MMF was associated with a greater probability of sinusoidal obstruction syndrome at expense of late‐onset forms, which totally resolved with treatment and no associated mortality was detected.

### 2.3 Conditioning regimen

The conditioning regimens used during the study period are detailed in Table S1. They were categorised according to the European Society for Blood and Marrow Transplantation classification and the Center for International Blood and Marrow Transplant Research consensus [13, 14] as follows: (1) myeloablative conditioning (MAC) (intravenous busulfan [Bu] dose >6.4 mg/kg or thiotepa dose of 10 mg/kg); and (2) reduced intensity conditioning (RIC) (intravenous Bu dose ≤6.4 mg/kg or thiotepa [TT] dose of 5 mg/kg). When two alkylating agents were combined, MAC was also defined if Bu dose of 6.4 mg/kg was used with thiotepa dose of 5 mg/kg.

Patients with Hodgkin's lymphoma always received Cy–Bu–Flu (CBF), whereas the rest of patients received CBF or TT–Bu–Flu (TBF) following institutional policies at the time of transplantation.

### 2.4 Graft‐versus‐host disease prophylaxis

Both GVHD prophylaxis schemas included cyclophosphamide (50 mg/kg/day) intravenously on days +3 and +4 and MMF at 1 g/8 h (orally or intravenously) from day +5 to day +35. Prior to 2017, CsA was also started on day +5, at a dose of 1.5 mg/kg/12 h intravenously, and then adjusted to achieve a therapeutic level of 200–300 ng/ml, finally converted to oral form until day +90. After 2017, sirolimus replaced CsA in order to improve posttransplantation outcomes. It was started on day +5 at a loading dose of 6 mg the first day and 4 mg daily onwards, with dose modification to achieve the targeted plasma levels between 8 and 16 ng/ml until day +90. Both CsA and sirolimus trough concentrations were monitored two times per week during the first month and at least once per week thereafter. In the absence of GVHD or relapse, CsA or sirolimus were gradually tapered from day +90 to day +150.

### 2.5 Supportive care

Patients were nursed in HEPA‐filtered rooms. Supportive care measures included ciprofloxacin (500 mg/12 h po) during neutropenia, *Pneumocystis jirovecii* prophylaxis with cotrimoxazol up to day +180, voriconazole (200 mg/12 h) until 2017 or posaconazole (300 mg/day) thereafter as antifungal prophylaxis from day +7 until day +100 or while on steroids to treat moderate to severe GVHD, and antiviral prophylaxis with oral acyclovir (800 mg/12 h) up to 1 year. Pre‐emptive antiviral therapy (PET) guided by quantitative real‐time PCR assays (QRT‐PCR) to prevent CMV disease was also used, as previously reported [15]. Epstein–Barr virus was routinely monitored during the first 6 months or if concomitant immunosuppression. *Adenovirus* was not consistently determined (only if persistent fever or organ dysfunction). Ursodeoxycholic acid (300 mg/8 h) was started on day −7 up to day +100. All patients received G‐CSF 5 mcg/kg/day from day +7 until absolute neutrophil count >1 × 10^9^/L for 3 consecutive days.

### 2.6 Definitions

Myeloid recovery was defined as the first day of an absolute neutrophil count of 0.5 × 109/L lasting for 3 consecutive days. Platelet recovery was defined as the first day of a platelet count of 20 × 109/L or higher, without transfusion support for 7 consecutive days. Patients who survived more than 28 days after transplantation and failed to achieve myeloid engraftment were considered to have primary graft failure. Acute GVHD (aGVHD) and chronic GVHD (cGVHD) were defined and graded according to standard criteria [16, 17]. SOS [18], CMV DNAemia and disease [19], EBV DNAemia [20], ADV DNAemia [21], mucositis [22] and BK polyomavirus‐associated haemorrhagic cystitis [23] were diagnosed according to consensus criteria. Toxicities were graded using NCI criteria (Common Toxicity Criteria version 5) with the exception of SOS and mucositis, which were graded using the new EBMT criteria [18] and the WHO classification [22], respectively. Hepatotoxicity was defined as an increase in alanine or aspartate aminotransferase above normal levels. Disease status, disease risk index (DRI) and haematopoietic cell transplantation‐specific comorbidity index (HCT‐CI) scores were calculated, as previously described [24, 25, 26].

### 2.7 Statistical analysis

Primary end point was overall survival (OS) and the cumulative incidence of GVHD. Secondary end points included nonrelapse mortality (NRM), relapse, event‐free survival (EFS), GVHD‐free, relapse‐free survival (GRFS) and other posttransplantation events (engraftment, SOS, CMV DNAemia, CMV‐related disease, EBV DNAemia, ADV DNAemia, oral mucositis, haemorrhagic cystitis, acute kidney injury [AKI], hepatotoxicity, thrombotic microangiopathy [TMA], hypercholesterolemia and hypertriglyceridemia). OS was calculated from the time of transplantation to death by any cause or to last follow‐up. EFS was calculated as survival from the time of transplantation without evidence of relapse or graft failure. GRFS was calculated as survival from the time of transplantation without evidence of relapse, graft failure, grades III–IV aGVHD or cGVHD requiring immunosuppressive treatment.

Patient and transplantation characteristics of both cohorts were compared using the chi‐square test with Yates’ correction and Fisher's exact test for categorical variables. Differences between medians were compared using the Mann–Whitney *U* test. Relapse, NRM, GVHD, and posttransplant toxicities were estimated by the cumulative incidence method [27, 28]. Univariate analyses of the association of clinical risk factors with these transplantation outcomes were calculated using the Gray test. Competing risks data were considered as follows: (a) for GVHD, relapse before GVHD or death; (b) for NRM, relapse; (c) for relapse, death with no previous relapse; or (d) for posttransplant events, death or relapse with no previous event. Time‐dependent covariates were analysed by univariate Cox regression models. When any time‐dependent covariate was included in the final models, multivariate analyses were performed by Cox proportional hazards regression [29], otherwise Fine and Gray test was used [30]. The probability of EFS, GRFS and OS were estimated from the time of transplantation using Kaplan–Meier curves, and univariate comparisons were done with the log‐rank test [31]. A value of *p* < .05 was used to determine statistical significance. Study variables included patient age, sex, prior autologous transplant, disease diagnosis, disease stage, DRI, HCT‐CI, CMV serostatus, type of conditioning regimen, source of stem cells, ABO blood group mismatch, CD34 and CD3 infused. Statistical analysis was conducted using R version 4.0.0 (the CRAN project).

## 3 RESULTS

### 3.1 Patients and transplant characteristics

Table 1 summarizes the characteristics of the 67 and 66 patients from the PTCy–CsA–MMF and PTCy–Sir–MMF cohorts, respectively. Patient, disease and transplant characteristics were similar in both cohorts, except for a higher median age in the latter (43 vs. 58 years; *p* < .001), who also received more often bone marrow as stem cell source (0% vs. 21%; *p* < .001) and myeloablative conditioning (48% vs. 74%; *p* < .001). The groups were otherwise well matched with respect to the remaining baseline characteristics.

**TABLE 1:** Patient, graft and transplantation‐related characteristics according to graft‐versus‐host disease (GVHD) prophylaxis

**Characteristics** **^a^**	**Entire cohort**	**PTCy–CsA–MMF**	**PTCy–Sir–MMF**	***p*‐Value**
Patients, no. (%)	133	67 (50)	66 (50)	
Age in years, median (range)	52 (18–71)	43 (18–67)	58 (22–71)	<.001
Male gender, no. (%)	82 (62)	42 (63)	40 (61)	.95
Weight in kilograms, median (range)	73 (47–138)	73 (47–138)	73 (49–125)	.34
Prior autologous transplant, no. (%)	52 (39)	28 (42)	24 (36)	.64
**Diagnosis**, no. (%)				.06
AML	36 (27)	17 (25)	19 (29)	
ALL	12 (9)	9 (13)	3 (5)	
MDS	10 (8)	4 (6)	6 (9)	
NHL	29 (22)	15 (22)	14 (21)	
HL	27 (20)	18 (27)	9 (14)	
CLL	4 (3)	2 (3)	2 (3)	
MM	5 (4)	0	5 (8)	
MPD	4 (3)	1 (1)	3 (5)	
MPD/MDS	4 (3)	0	4 (6)	
**Disease stage at transplant**, no. (%)				.05
Early	42 (32)	21 (31)	21 (32)	
Intermediate	52 (39)	32 (48)	20 (30)	
Advanced	39 (29)	14 (21)	25 (38)	
**DRI**, no. (%)				.31
Low	37 (28)	16 (24)	21 (32)	
Intermediate	61 (46)	32 (48)	29 (44)	
High	33 (25)	19 (28)	14 (21)	
Very high	2 (2)	0	2 (3)	
**HCT‐CI**, no. (%)				.61
0	35 (26)	21 (31)	14 (21)	
1−2	31 (23)	14 (21)	17 (26)	
≥3	67 (50)	32 (48)	35 (53)	
**Donor age in years**, median (range)	40 (7–72)	41 (7–72)	39 (15–67)	.34
**Female donor to male recipient**, no. (%)	32 (24)	19 (28)	13 (20)	.33
**CMV status**, no. (%)				.07
Donor−/recipient−	10 (8)	3 (5)	7 (11)	
Donor−/recipient+	18 (14)	8 (12)	10 (15)	
Donor+/recipient−	15 (11)	12 (18)	3 (5)	
Donor+/recipient+	90 (68)	44 (66)	46 (70)	
**ABO blood group mismatch**, no. (%)				.23
None	91 (68)	42 (63)	49 (74)	
Minor	24 (18)	13 (19)	11 (17)	
Major	15 (11)	9 (13)	6 (9)	
Bidirectional	3 (2)	3 (4)	0	
**Source of stem cells**, no. (%)				<.001
Bone marrow	14 (11)	0	14 (21)	
Peripheral blood	119 (89)	67 (100)	52 (79)	
**Conditioning regimen**, no. (%)				<.001
Myeloablative	81 (61)	32 (48)	49 (74)	
TBF	71 (53)	22 (33)	49 (74)	
CBF	10 (8)	10 (15)	0	
Reduced intensity	52 (39)	35 (52)	17 (26)	
TBF	1 (1)	0	1 (2)	
CBF	51 (38)	35 (52)	16 (24)	
**Cells infused**, median (range)				
CD34 × 10^6^/kg	6.4 (1.4–15.4)	6.2 (2.2–15.4)	6.9 (1.4–13.8)	.34
CD3 × 10^8^/kg	221 (20–600)	237 (86–541)	195 (20–600)	.04
**Follow‐up in days**, median (range)	761 (95–2536)	1486 (676–2536)	411 (95–1033)	<.001

Abbreviations: ALL, acute lymphoid leukaemia; AML, acute myeloid leukaemia; CBF, cyclophosphamide, busulfan and fludarabine; CLL, chronic lymphoid leukaemia; CMV, cytomegalovirus; DRI, disease‐risk index; HCT‐CI, haematopoietic cell transplant‐comorbidity index; HL, Hodgkin lymphoma; MDS, myelodysplatic syndrome; MM, multiple myeloma; MPD, myeloproliferative disease; MPD/MDS, myeloproliferative/myelodysplatic neoplasm; NHL, non‐Hodgkin lymphoma; PTCy–CsA–MMF, posttransplant cyclophosphamide, cyclosporine and mycophenolate; PTCy–Sir–MMF, posttransplant cyclophosphamide, sirolimus and mycophenolate; TBF, thiotepa, busulfan and fludarabine.

^a^ Percentages may not sum to 100 because of rounding.

### 3.2 Neutrophil engraftment

In the PTCy–CsA–MMF cohort, two patients died on days 13 and 25 after stem cell infusion without evidence of myeloid engraftment. One additional patient with initial neutrophil recovery had secondary graft failure. The remaining 64 patients achieved stable neutrophil engraftment at a median time of 18 days (range 13−34). In the PTCy–Sir–MMF cohort, one patient died on day 14 after stem cell infusion without evidence of myeloid engraftment. One patient with myelofibrosis had a primary graft failure and underwent a second allogeneic SCT and one additional patient had secondary graft failure. The remaining 63 patients achieved stable neutrophil engraftment at a median time of 19 days (range 2−38).

**FIGURE 1:**
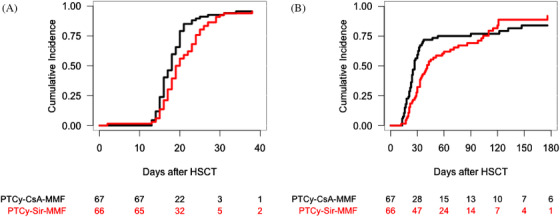
Cumulative incidence of neutrophil recovery according to the graft‐versus‐host disease (GVHD) prophylaxis (A). Cumulative incidence of platelet recovery according to the GVHD prophylaxis (B)

The cumulative incidence of sustained neutrophil recovery at 40 days in the PTCy–CsA–MMF and PTCy–Sir–MMF cohorts was 94% (95% CI, 88–100) and 95% (95% CI, 90–100), respectively (*p* = .08) (Figure 1A) (Table 2).

**TABLE 2:** Transplantation outcomes according to graft‐versus‐host disease prophylaxis

Outcome	Entire cohort	PTCy–CsA–MMF	PTCy–Sir–MMF	*p*‐Value
**Myeloid engraftment**, cumulative incidence at d +40 (% [95% CI])	95 (91–99)	94 (88–100)	95 (90–100)	.08
Median time in days, median (range)	18 (2–38)	18 (13–34)	19 (2–38)	
**Platelet engraftment**, cumulative incidence at d +180 (% [95% CI])	87 (81–94)	83 (74–93)	92 (84–100)	.19
Median time in days, median (range)	29 (12–319)	24 (13–319)	35 (12–176)	
**aGVHD grades II–IV**, cumulative incidence at d +100 (% [95% CI])	38 (30–46)	46 (34–58)	29 (18–40)	.05
Median time in days, median (range)	30 (14–114)	29 (14–99)	30 (16–114)	
**aGVHD grades III–IV**, cumulative incidence at d +100 (% [95% CI])	12 (7–18)	12 (4–20)	12 (4–20)	.79
Median time in days, median (range)	31 (15–114)	23 (15–99)	34 (25–114)	
**cGVHD,** 2‐year cumulative incidence (% [95% CI])	49 (39–59)	42 (30–55)	61 (44–77)	.18
Median time in days, median (range)	177 (60–1328)	173 (97–1328)	184 (60–576)	
**cGVHD moderate to severe,** 2‐year cumulative incidence (% [95% CI])	27 (18–35)	27 (16–38)	23 (11–35)	.84
Median time in days, median (range)	173 (87–1328)	164 (97–1328)	184 (87–576)	
**NRM**, 2‐year cumulative incidence (% [95% CI])	27 (19–35)	24 (14–34)	29 (18–40)	.38
Median time in days, median (range)	91 (13–728)	106 (13–728)	82 (14–300)	
**Relapse,** 2‐year cumulative incidence (% [95% CI])	19 (11–26)	24 (14–34)	10 (2–19)	.07
Median time in days, median (range)	232 (62–1153)	277 (71–1153)	148 (62–347)	
**EFS,** 2‐year cumulative incidence (% [95% CI])	52 (43–61)	51 (40–64)	61 (49–75)	.55
**OS,** 2‐year cumulative incidence (% [95% CI])	59 (50–68)	58 (47–71)	64 (52–78)	.96
**GRFS,** 2‐year cumulative incidence (% [95% CI])	40 (31–49)	39 (29–52)	42 (30–59)	.82

Abbreviations: aGVHD, acute graft‐versus‐host disease; cGVHD, chronic GVHD; CI, confidence interval; EFS, event‐free survival; GRFS, GVHD‐free, relapse‐free survival.; NRM, nonrelapse mortality; OS, overall survival; PTCy–CsA–MMF, posttransplant cyclophosphamide, cyclosporine and mycophenolate; PTCy–Sir–MMF, posttransplant cyclophosphamide, sirolimus and mycophenolate.

### 3.3 Platelet engraftment

Nine of 64 patients with myeloid engraftment in the PTCy–CsA–MMF cohort died between 75 and 398 days after transplantation without platelet recovery. The remaining 55 patients had platelet engraftment at a median time of 24 days (range 13–319). Nine of 63 patients with myeloid engraftment in the PTCy–Sir–MMF cohort died between 45 and 241 days after transplantation without platelet recovery. The remaining 54 patients had platelet engraftment at a median time of 35 days (range 12–176).

The cumulative incidence of sustained platelet engraftment at 180 days in the PTCy–CsA–MMF and PTCy–Sir–MMF cohorts was 83% (95% CI, 74–93) and 92% (95% CI, 84–100), respectively (*p* = 0.19) (Figure 1B) (Table 2).

### 3.4 Acute GVHD

In the PTCy–CsA–MMF cohort, 40 patients (59%) developed aGVHD at a median time of 30 days (range 11–99): grade I in nine (13%), grade II in 23 (34%), grade III in four (6%) and grade IV in four (6%). In the PTCy–Sir–MMF cohort, 37 patients (57%) developed aGVHD at a median time of 32 days (range 15–114): grade I in 17 (26%), grade II in 11 (17%), grade III in three (5%) and grade IV in six (9%).

**FIGURE 2:**
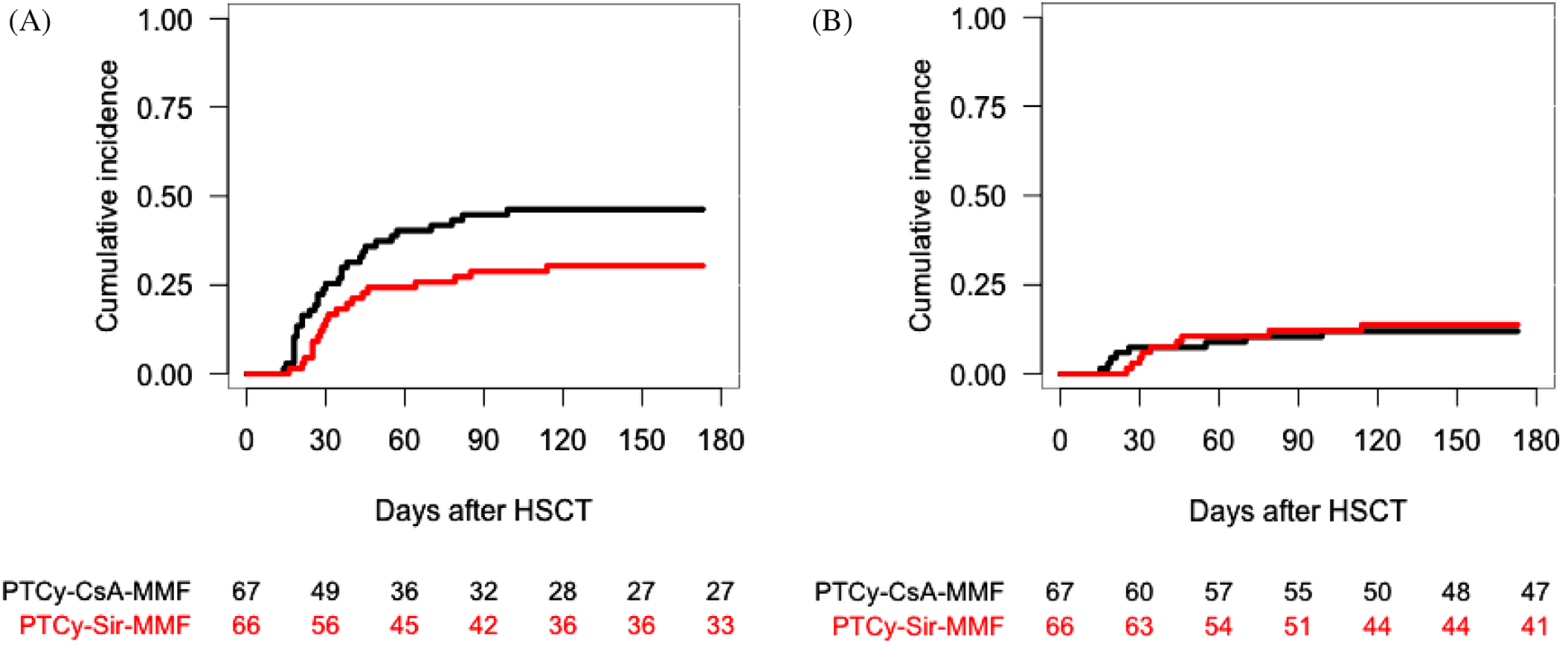
Cumulative incidence of grades II–IV acute graft‐versus‐host disease (aGVHD) according to the GVHD prophylaxis (A). Cumulative incidence of grades III–IV aGVHD according to the GVHD prophylaxis (B)

The cumulative incidence of aGVHD grades II–IV at 100 days in the PTCy–CsA–MMF and PTCy–Sir–MMF cohorts was 46% (95% CI, 34–58) and 29% (95% CI, 18–40), respectively (*p* = .05) (Figure 2A), whereas for grades III–IV it was 12% (95% CI, 4–20) and 12% (95% CI, 4–20), respectively (*p* = .79) (Figure 2B) (Table 2).

### 3.5 Chronic GVHD

In the PTCy–CsA–MMF cohort, 27 of 59 patients at risk (46%) developed cGVHD at a median time of 173 days (range 97–1328), with 10 patients (17%) being mild, eight (14%) moderate, and nine (15%) severe cGVHD. In the PTCy–Sir–MMF cohort, 26 of 53 patients at risk (49%) developed cGVHD at a median time of 184 days (range 60–576), with 14 patients (26%) being mild, six (11%) moderate and six (11%) severe cGVHD.

**FIGURE 3:**
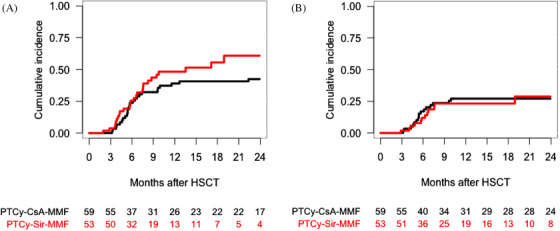
Cumulative incidence of chronic graft‐versus‐host disease (cGVHD) according to the GVHD prophylaxis (A). Cumulative incidence of moderate to severe cGVHD according to the GVHD prophylaxis (B)

The 2‐year cumulative incidence of any cGVHD in the PTCy–CsA–MMF and PTCy–Sir–MMF cohorts was 42% (95% CI, 30–55) and 61% (95% CI, 44–77), respectively (*p* = .18) (Figure 3A), whereas it was 27% (95% CI, 16–38) and 23% (95% CI, 11–35) (*p* = .84), respectively, of moderate to severe cGVHD (Table 2) (Figure 3B).

### 3.6 NRM and causes of death

Sixteen patients (24%) in the PTCy–CsA–MMF cohort died without prior relapse at a median time of 94 days (range 13–728), whereas in the PTCy–Sir–MMF cohort, 18 patients (27%) died without prior relapse at a median time of 89 days (range 14−300). Causes of death in the different platforms are shown in Table 3.

**TABLE 3:** Causes of death according to graft‐versus‐host disease prophylaxis

**Cause**	**PTCy–CsA–MMF (*N* = 30)**	**PTCy–Sir–MMF (*N* = 21)**
**Relapse**, no.	14	3
**Graft‐versus‐host disease**, no.	8	10
**Infection**, no.	4^a^	4^b^
**Sinusoidal obstruction syndrome**, no.	2	–
**Secondary solid cancer**, no.	2	–
**Graft failure**, no.	–	1
**Haemorrhage**, no.	–	1
**Bowel ischemia**, no.	–	1
**Unknown**, no.	–	1

Abbreviations: PTCy–CsA–MMF, posttransplant cyclophosphamide, cyclosporine and mycophenolate; PTCy–Sir–MMF, posttransplant cyclophosphamide, sirolimus and mycophenolate.

^a^ One nondocumented infection, one disseminated *Candida parapsilosis* infection, one respiratory syncytial virus pneumonia, one disseminated adenovirus infection.

^b^ One nondocumented infection, one *Pseudomonas aeruginosa* pneumonia, one human herpesvirus 6 encephalitis, one mixed infection (CMV infection, invasive pulmonary aspergillosis and septic shock by *Staphilococus haemolyticus*).

**FIGURE 4:**
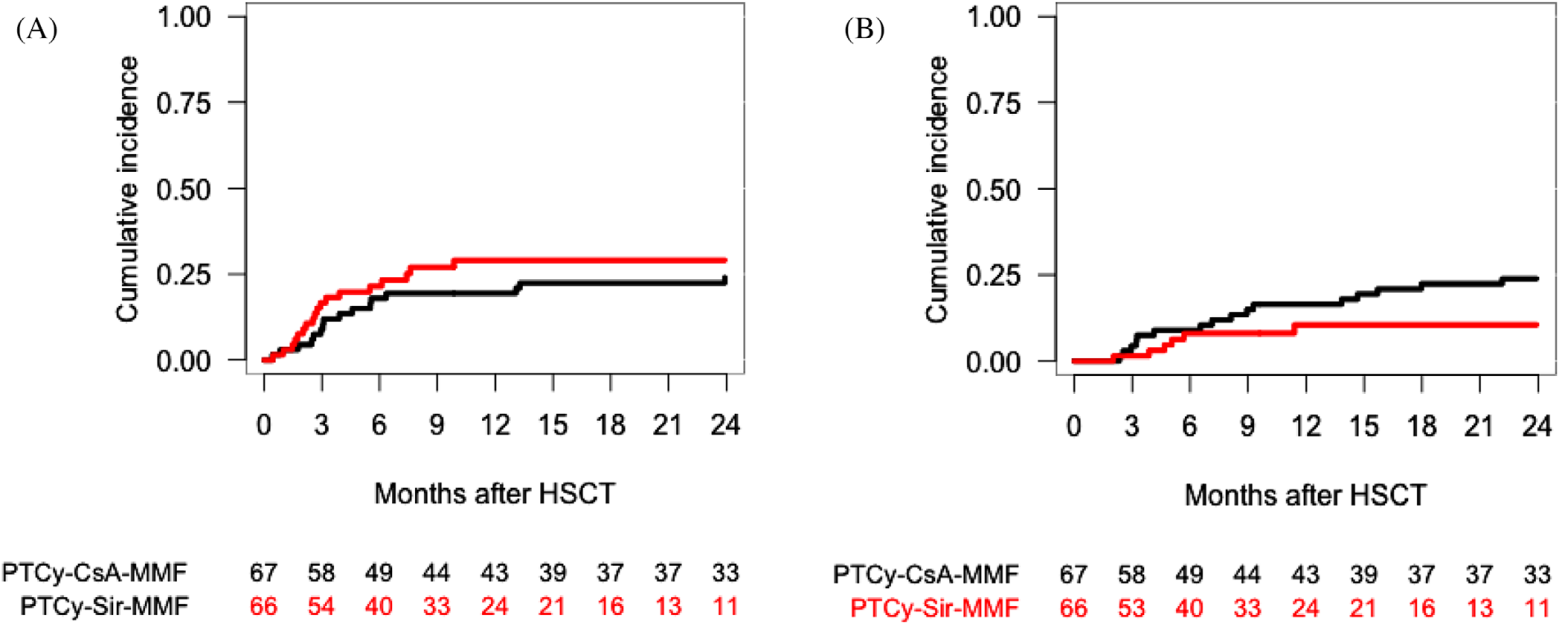
Cumulative incidence of overall nonrelapse mortality according to the graft‐versus‐host disease (GVHD) prophylaxis (A). Cumulative incidence of relapse according to the GVHD prophylaxis (B)

The 2‐year cumulative incidence of NRM in the PTCy–CsA–MMF and PTCy–Sir–MMF cohorts was 24% (95% CI, 14−34%) and 29% (95% CI, 18−40%), respectively (*p* = .38) (Table 2) (Figure 4A).

### 3.7 Relapse

Twenty patients (30%) in the PTCy–CsA–MMF cohort and six patients (9%) in the PTCy–Sir–MMF cohort relapsed at a median time of 277 days (range 71−1153) and 148 days (range 62–347), respectively.

The 2‐year cumulative incidence of relapse in the PTCy–CsA–MMF and PTCy–Sir–MMF cohorts was 24% (95% CI, 14−34%) and 10% (95% CI, 2−19%), respectively (*p* = .07) (Table 2) (Figure 4B). We tested the impact of GVHD prophylaxis on relapse within diagnosis subgroups and conditioning intensity, with no significant differences between subgroups.

### 3.8 Survival outcomes

Thirty‐seven patients (55%) in the PTCy–CsA–MMF cohort and 45 patients (68%) in the PTCy–Sir–MMF cohort remained alive at a median follow‐up of 1486 days (range 676−2536) and 411 days (range 95–1033), respectively.

**FIGURE 5:**
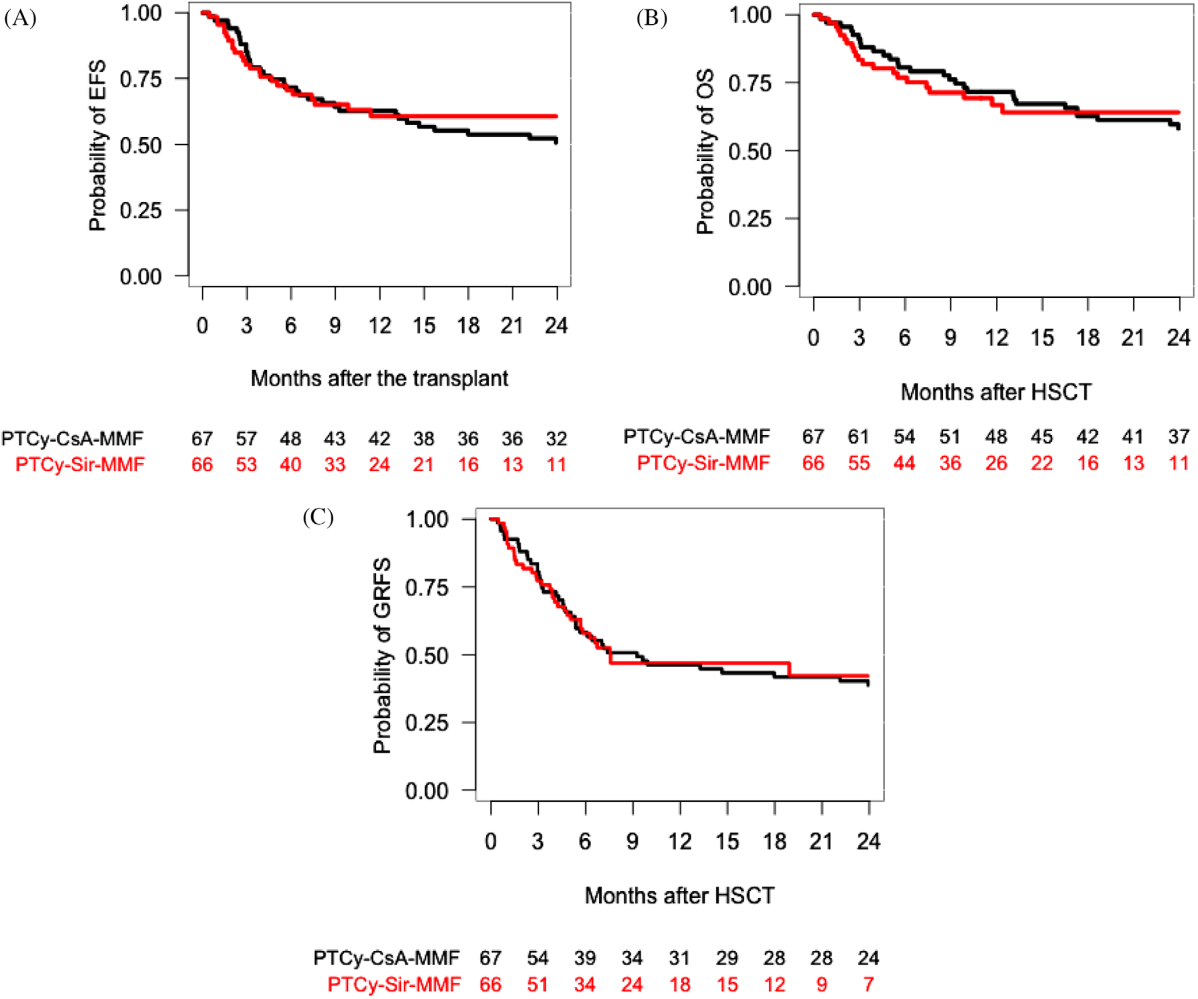
One‐year overall event‐free survival according to the graft‐versus‐host disease (GVHD) prophylaxis (A). One‐year overall survival according to the GVHD prophylaxis (B). One‐year overall GVHD‐free and relapse‐free survival according to the GVHD prophylaxis (C)

According to GVHD prophylaxis, EFS, OS and GRFS at 2 years was 51% (95% CI, 40−64%), 58% (95% CI, 47−71%) and 39% (95% CI, 29−52%) for PTCy–CsA–MMF, and 61% (95% CI, 49−75%), 64% (95% CI, 52−78%) and 42% (95% CI, 30−59%) for PTCy–Sir–MMF, respectively (*p* = .55, .96 and .82) (Table 2) (Figure 5).

### 3.9 Posttransplant events

Posttransplant events according to GVHD prophylaxis are detailed in Table 4.

**TABLE 4:** Posttransplant events according to graft‐versus‐host disease prophylaxis

Outcome	Entire cohort	PTCy–CsA–MMF	PTCy–Sir–MMF	*p*‐Value
**CMV DNAemia requiring PET**, 1‐year cumulative incidence [% (95% CI])	54 (46–63)	51 (39–63)	58 (46–70)	.25
Median time in days, median (range)	48 (1–234)	50 (4–234)	46 (1–101)	
**CMV‐related disease**, 1‐year cumulative incidence (% [95% CI])	6 (2–10)	9 (2–16)	3 (0–7)	.16
Median time in days, median (range)	78 (44–127)	80 (44–127)	78 (76–81)	
**EBV DNAemia,** 1‐year cumulative incidence (% [95% CI])	16 (10–23)	16 (8–25)	16 (7–25)	.85
Median time in days, median (range)	104 (6–424)	111 (15–424)	101 (6–203)	
**ADV requiring cidofovir,** 1‐year cumulative incidence (% [95% CI])^a^	2 (0–14)	0	3 (0–7)	.15
**BKPyV‐HC**, cumulative incidence at d +100 (% [95% CI])	41 (32–49)	33 (22–44)	48 (36–61)	.13
Median time in days, median (range)	30 (7–127)	30 (7–127)	30 (13–82)	
**BKPyV‐HC III–IV**, cumulative incidence at d +100 (% [95% CI])	15 (9–21)	10 (3–18)	20 (10–29)	.15
**SOS**, CI at d +100 (% [95% CI])	9 (4–14)	4 (0–9)	14 (5–22)	.08
Median time in days, median (range)	26 (4–84)	13 (4–19)	26 (14–84)	
**Oral mucositis**, cumulative incidence at d +30 (% [95% CI])	53 (45–62)	48 (36–60)	59 (47–71)	.52
Median time in days, median (range)	8 (1–14)	4 (4–9)	8 (1–14)	
**Oral mucositis III–IV**, cumulative incidence at d +30 (% [95% CI])	11 (5–16)	3 (0–7)	18 (9–27)	.005
**Acute kidney injury**, cumulative incidence at d +180 (% [95% CI])	45 (36–53)	57 (45–69)	32 (21–44)	.003
Median time in days, median (range)	44 (1–348)	19 (2–96)	60 (22–143)	
**Acute kidney injury III–IV**, cumulative incidence at d +180 (% [95% CI])	8 (3–12)	11 (3–18)	5 (0–10)	.2
**Thrombotic microangiopathy**, cumulative incidence at d +180 (% [95% CI])	10 (5–15)	18 (9–27)	2 (0–4)	.002
Median time in days, median (range)	82 (42–140)	82 (42–140)	^b^	
**Hepatotoxicity**, cumulative incidence at d +180 (% [95% CI])	76 (69–83)	67 (56–78)	85 (76–93)	.21
Median time in days, median (range)	33 (1–318)	39 (1–318)	24 (1–179)	
**Hepatotoxicity III–IV**, cumulative incidence at d +180 (% [95% CI])	28 (20–36)	32 (20–43)	24 (13–35)	.05
**Hypercholesterolemia**, cumulative incidence at d +180 (% [95% CI])	45 (37–54)	34 (23–46)	56 (44–68)	.01
Median time in days, median (range)	83 (14–344)	83 (14–337)	83 (20–344)	
**Hypercholesterolemia III–IV**, cumulative incidence at d +180 (% [95% CI])	4 (1–7)	5 (0–10)	3 (0–7)	.34
**Hypertriglyceridemia**, cumulative incidence at d +180 (% [95% CI])	84 (78–90)	81 (71–90)	88 (80–96)	.71
Median time in days, median (range)	39 (1–183)	35 (6–183)	44 (1–167)	
**Hypertriglyceridemia III–IV**, cumulative incidence at d +180 (% [95% CI])	17 (11–24)	18 (9–28)	17 (7–26)	.73

Abbreviations: ADV, adenovirus; BKPyV‐HC, BK polyomavirus‐associated haemorrhagic cystitis; CI, confidence interval; CMV, cytomegalovirus; EBV, Epstein–Barr virus; PTCy–CsA–MMF, posttransplant cyclophosphamide, cyclosporine and mycophenolate; PTCy–Sir–MMF, posttransplant cyclophosphamide, sirolimus and mycophenolate; SOS, sinusoidal obstruction syndrome.

^a^ ADV not evaluated in 25 and 12 patients with PTCy–CsA–MMF and PTCy–Sir–MMF, respectively.

^b^ Only one patient with PTCy–Sir–MMF developed thrombotic microangiopathy 83 days after transplantation.

#### 3.9.1 Viral infections

CMV infection requiring PET occurred in 33 recipients in the PTCy–CsA–MMF cohort and in 38 patients in the PTCy–Sir–MMF cohort. CMV‐related disease was documented in six and two patients in the PTCy–CsA–MMF and PTCy–Sir–MMF cohorts, respectively (one patient with gastrointestinal involvement had no prior detectable CMV DNAemia).

EBV DNAemia was detected in 12 recipients in the PTCy–CsA–MMF cohort and in 10 patients in the PTCy–Sir–MMF cohort. It spontaneously resolved in 17 patients, whereas the remaining five (PTCy–CsA–MMF = 4, PTCy–Sir–MMF = 1) were treated and resolved with rituximab. Cases of EBV‐associated lymphoproliferative disease were not observed.

ADV DNAemia requiring cidofovir occurred in two patients at 59 and 69 days after transplantation, both in the PTCy–Sir–MMF cohort. One patient with gastrointestinal involvement resolved after discontinuation of immunosuppression and the remaining one had a fatal encephalitis in the context of refractory GVHD.

Two and three patients developed early‐onset haemorrhagic cystitis in the PTCy–CsA–MMF and PTCy–Sir–MMF cohorts, respectively. BK polyomavirus‐associated haemorrhagic cystitis was detected in 23 recipients in the PTCy–CsA–MMF cohort and in 32 patients in the PTCy–Sir–MMF cohort. BK polyomavirus‐associated haemorrhagic cystitis is detailed in Table S2.

#### 3.9.2 Toxicities

In univariate analysis, there was a trend towards a higher cumulative incidence of SOS in patients with PTCy–Sir–MMF (14% vs. 4%; *p* = .08), grades III–IV oral mucositis and hypercholesterolemia, whereas patients with PTCy–CsA–MMF showed a significantly higher cumulative incidence of AKI (57% vs. 32%; *p* = .003), TMA (18% vs. 2%; *p* = .002) and grades III–IV hepatotoxicity (32% vs. 24%; *p* = .05). No differences regarding hypertriglyceridemia were detected. Details of toxicities are described in Table S2.

Three patients in the PTCy–CsA–MMF cohort developed classical SOS at a median follow‐up of 13 days (range 4−19), whereas nine patients in the PTCy–Sir–MMF developed SOS (classical and late‐onset SOS in two and seven patients, respectively) at a median time of 26 days (range 14−84). One patient with late‐onset SOS resolved with diuretics and fluid restriction, whereas the remaining 11 patients required defibrotide therapy. All nine patients with PTCy–Sir–MMF resolved and none of them required discontinuation of sirolimus, whereas two of the patients with classical SOS and PTCy–CsA–MMF died (one patient with myelofibrosis and one patient with acute lymphoblastic leukaemia who had previously received inotuzumab).

### 3.10 Multivariate analyses for different outcomes and posttransplant events

In multivariate analysis, PTCy–CsA–MMF was associated with a higher probability of AKI (HR 2.1, 95% CI, 1.21–3.57, *p* = .008) and larger risk of TMA (HR 12.5, 95% CI, 1.66–93.5, *p* = .014), whereas PTCy–Sir–MMF was associated with a greater probability of SOS (HR 10.8, 95% CI, 1.52–77, *p* = .018). Details of statistical analyses are described in Table S3. None of these variables, including subgroup analyses of AKI grades, had a statistically significant impact on OS or EFS when analysed by Cox proportional‐hazards regression model.

## 4 DISCUSSION

In this study, we found that a CNI‐free GVHD prophylaxis with PTCy–Sir–MMF provided similar rates of engraftment, GVHD, NRM, OS and GRFS when compared with a classical CsA‐based approach with PTCy–CsA–MMF. Patients in the PTCy–Sir–MMF cohort had a significantly lower toxicity in terms of AKI and TMA. In contrast, a higher incidence of late‐onset SOS was observed in the sirolimus‐containing approach, but all these SOS cases resolved.

We acknowledge the limitations of our study: (i) retrospective nature; (ii) nonrandomised comparison; (iii) small sample size; and (iv) short follow‐up in the PTCy–Sir–MMF cohort. Despite these limitations, this is, to our knowledge, the first study comparing a CNI‐free with a CNI‐based GVHD prophylaxis in a real‐life unselected population of patients undergoing haploidentical transplantation for a variety of haematologic malignancies according to previously established criteria for donor selection, conditioning regimen and stem cell source. In addition, patients in the two cohorts were comparable in most baseline characteristics, except for a higher median age in the PTCy–Sir–MMF group, who also received more often bone marrow as stem cell source and myeloablative conditioning.

The low incidence of aGVHD grades III–IV observed, 12% at 100 days with both GVHD prophylaxis approaches, confirms the efficacy reported in a few small noncomparative studies with PTCy and sirolimus prophylaxis in patients undergoing SCT from HLA‐matched related or unrelated donors [7, 9], as well as in recipients of grafts from HLA‐haploidentical donors [10, 11]. To further reduce the rate of aGVHD using PTCy–Sir–MMF, we have recently replaced peripheral blood with bone marrow as stem cell source. Interestingly, none of the 14 bone marrow transplant patients has yet developed grades III–IV aGVHD (data not shown). These encouraging results should be confirmed in a larger series of patients. Regarding the incidence of moderate to severe cGVHD in the sirolimus‐containing prophylaxis cohort (23%), we also confirmed the low rate previously reported by Cieri et al. and Bejanyan et al. in a similar haplo‐SCT cohort (20% and 18.8%, respectively).

In contrast with prior studies [7, 9, 11], in which the cumulative incidence of NRM at 1 year ranged from 13% to 17%, in our series it was 24%, with no significant differences between the two prophylactic approaches. Solomon et al. did not included haplo‐SCT and only used RIC regimens, whereas the San Raffaele group used treosulfan instead of Bu. Recently, Bejanyan et al. has shown similar rates of NRM (18.8% at 1 year) in a younger cohort of haplo‐SCT recipients with pharmacokinetically targeted Bu [10]. Our data may reflect an unbalanced proportion of patients with pejorative prognostic factors in an unselected series, highlighting that all patients received a Bu‐based conditioning regimen, the substantial proportion of MAC (61%) or who had previously undergone an autologous transplant (39%) and the higher proportion of patients with HCT‐CI ≥3 (50%). The significantly older age of the patients who received PTCy–Sir–MMF may also have an impact on NRM.

The apparent decrease in the cumulative incidence of relapse achieved in the sirolimus‐treated group (10% at 2 years), not only compared to the group treated with CsA in our study, but also to that reported in other studies using a similar CNI‐free prophylaxis [7, 9, 10, 11], should be interpreted cautiously. The higher proportion of patients who received myeloablative conditioning in the CNI‐free prophylaxis group, which may lead to a lower relapse rate [32], could explain, at least in part, these encouraging results. A larger sample and longer follow‐up could establish the relative role of GVHD conditioning and prophylaxis in these and other survival outcomes.

Conflicting results have been reported regarding an increased risk of CMV infection in haplo‐SCT [12, 33, 34], as well as an increased incidence of CMV‐related disease in this setting [33, 35]. We have confirmed that approximately half of the patients in our study had CMV infection requiring PET, but the rate of CMV‐related disease in the PTCy–Sir–MMF group remained very low (3%), despite the significant proportion of older patients in this group, which has been previously described as an independent risk factor for CMV infection [34].

Regarding toxicity, it should be noted that the cohort with PTCy–Sir–MMF prophylaxis had a lower incidence of AKI and TMA, but that of SOS increased, especially late‐onset forms (seven out of nine patients), which typically manifested as fluid retention and hepatitis without hyperbilirubinemia. All of them resolved and none of the patients needed discontinuation of sirolimus. Of note, the two SOS‐related deaths were detected, both in the PTCy–CsA–MMF subgroup. The use of sirolimus has been associated with larger risk of SOS in the setting of CNI‐based GVHD prophylaxis [36], although previous experience in the context of CNI‐free regimens does not support this association [7, 10, 11]. The higher incidence of SOS in our study could be related to our target of sirolimus levels, which are higher than previous studies [7, 11], following prior evidence of a lower incidence of CMV [8]. To note, Bejanyan et al. used similar target of sirolimus, showing a lower rate of SOS. This study involves a younger cohort of patients with a lower proportion of lymphoproliferative diseases. The use of pharmacokinetically targeted Bu could also explain this phenomenon [10]. The analysis of sirolimus through levels and its role in SOS is extremely challenging. Sirolimus is largely cleared by the liver via the cytochrome P450 system (CYP3A4), and when liver dysfunction occurs sirolimus levels increase as its clearance is significantly reduced [37]. As previously suggested [12], replacing busulfan with treosulfan or establishing busulfan levels monitoring is now under consideration in our institutions in order to decrease this complication [38, 39].

In conclusion, haplo‐SCT with PTCy–Sir–MMF as GVHD prophylaxis provides similar outcomes to those with PTCy–CsA–MMF. In terms of toxicity, while CNI‐free prophylaxis produced less renal toxicity and microangiopathy, an increase in late‐onset SOS was also observed, all of which resolved with treatment without sirolimus discontinuation. Prospective randomised studies comparing GVHD prophylaxis schemas are warranted in order to confirm these findings.

## CONFLICT OF INTEREST

The authors declare that there is no conflict of interest.

## AUTHOR CONTRIBUTIONS

Rafael Hernani, José Luis Piñana and Jaime Sanz conceived the study and interpreted the data, wrote the paper and performed the statistical analyses. Ariadna Pérez, Abdiel Quintero, Juan Montoro, Juan C. Hernández‐Boluda, Carlos Carretero, Aitana Balaguer‐Roselló, Manuel Guerreiro, Ignacio Lorenzo, Cristóbal Aguilar, Estela Giménez, David Navarro, Miguel A. Sanz and Carlos Solano reviewed the paper and contributed to the final draft.

## Supporting information

Supporting Information
