# Supplementary material for: Sirolimus versus cyclosporine in haploidentical stem cell transplantation with posttransplant cyclophosphamide and mycophenolate mofetil as graft‐versus‐host disease prophylaxis
Source: EJHaem. 2021 Mar 18;2(2):236–48. doi: 10.1002/jha2.183 (PMC9175741; doi:10.1002/jha2.183)
Supplement: Supplementary file 1 — Supporting Information [file JHA2-2-236-s001.docx]

**Supplementary file**

**Supplementary tables**

Table S1. Conditioning regimens and total dose of chemotherapy

|  | **Thiotepa** | **Cyclophosphamide** | **Busulfan** | **Fludarabine** |
| --- | --- | --- | --- | --- |
| **MAC** |  |  |  |  |
| T2B3F | 10 mg/kg |  | 9.6 mg/kg | 150 mg/m2 |
| T2B2F | 10 mg/kg |  | 6.4 mg/kg | 150 mg/m2 |
| T2B1F | 10 mg/kg |  | 3.2 mg/kg | 150 mg/m2 |
| T1B2F | 5 mg/kg |  | 6.4 mg/kg | 150 mg/m2 |
| CB4F |  | 29 mg/kg | 12.8 mg/kg | 160 mg/m2 |
| CB3F |  | 29 mg/kg | 9.6 mg/kg | 150 mg/m2 |
| **RIC** |  |  |  |  |
| T1B1F | 5 mg/kg |  | 3.2 mg/kg | 150 mg/m2 |
| CB2F |  | 29 mg/kg | 6.4 mg/kg | 150 mg/m2 |
| CB1F |  | 29 mg/kg | 3.2 mg/kg | 150 mg/m2 |

Abbreviations, TBF: thiotepa/busulfan/fludarabine; CBF: cyclophosphamide/busulfan/fludarabine; MAC: mieloablative conditioning; RIC: reduced-intensity regimen.

Table S2. Grade of toxicities and BKPyV-haemorrhagic cystitis according to graft-versus-host disease prophylaxis

| **Toxicity** * | **PTCy-CsA-MMF** | **PTCy-Sir-MMF** |
| --- | --- | --- |
| **BKPyV-haemorrhagic cystitis**, no. (%) | 23 (34) | 32 (48) |
| Grade 1 – 2 | 16 (24) | 19 (29) |
| Grade 3 – 4 | 7 (10) | 13 (20) |
| **SOS**, no. (%) | 3 (4) | 9 (14) |
| Mild | 0 | 0 |
| Moderate | 0 | 4 (6) |
| Severe | 2 (3) | 1 (1) |
| Very severe | 1 (1) | 4 (6) |
| **Oral mucositis**, no. (%) | 32 (48) | 39 (59) |
| Grade 1 – 2 | 30 (45) | 27 (41) |
| Grade 3 – 4 | 2 (3) | 12 (18) |
| **Acute kidney injury**, no. (%) | 45 (67) | 22 (33) |
| Grade 1 – 2 | 38 (57) | 20 (30) |
| Grade 3 – 4 | 7 (10) | 2 (3) |
| **Thrombotic microangiopathy**, no. (%) | 12 (18) | 1 (1) |
| **Hepatotoxicity**, no. (%) | 60 (90) | 60 (91) |
| Grade 1 – 2 | 32 (48) | 45 (68) |
| Grade 3 – 4 | 28 (42) | 15 (23) |
| **Hypercholesterolemia**, no. (%) | 30 (45) | 44 (67) |
| Grade 1 – 2 | 25 (37) | 42 (64) |
| Grade 3 – 4 | 5 (7) | 2 (3) |
| Data not available | 2 (3) | 4 (6) |
| **Hypertriglyceridemia**, no. (%) | 55 (82) | 58 (88) |
| Grade 1 – 2 | 43 (64) | 48 (73) |
| Grade 3 – 4 | 12 (18) | 10 (15) |
| Data not available | 2 (3) | 3 (5) |

* Percentages may not sum to 100 because of rounding.

Abbreviations, PTCy-CsA-MMF: postransplant cyclophosphamide, cyclosporine and mycophenolate; PTCy-Sir-MMF: postransplant cyclophosphamide, sirolimus and mycophenolate; BKPyV: BK polyomavirus; SOS: sinusoidal obstruction syndrome.

Table S3. Univariate and multivariate analyses of AKI, thrombotic microangiopathy and SOS

|  |  | **Univariate analysis** | | **Multivariate analysis** | |
| --- | --- | --- | --- | --- | --- |
| **Outcome** | **Variable** | **CumInc (%)*** | **P** | **HR (95% CI)** | **P** |
| **AKI** |  |  |  |  |  |
|  | D/R sex combination |  | 0.024 |  | 0.035 |
|  | Female D / Male R | 62 (46 – 79) |  | 1.82 (1.04 – 3.18) |  |
|  | Other | 39 (29 – 48) |  | 1 |  |
|  | Stem-cell source |  | 0.09 |  |  |
|  | Peripheral blood | 47 (38 – 56) |  |  |  |
|  | Bone marrow | 21 (0 – 43) |  |  |  |
|  | Conditioning regimen |  | 0.053 |  |  |
|  | CBF | 52 (40 – 65) |  |  |  |
|  | TBF | 38 (27 – 49) |  |  |  |
|  | GVHD prophylaxis |  | 0.003 |  | 0.008 |
|  | PTCy-CsA-MMF | 57 (45 – 69) |  | 2.1 (1.21 – 3.57) |  |
|  | PTCy-Sir-MMF | 32 (21 – 44) |  | 1 |  |
| **Thrombotic microangiopathy** |  |  |  |  |  |
|  | Sex |  | 0.078 |  |  |
|  | Female | 16 (6 – 26) |  |  |  |
|  | Male | 6 (0 – 11) |  |  |  |
|  | Prior auto-SCT |  | 0.02 |  | 0.004 |
|  | Yes | 17 (7 – 28) |  | 3.35 (1.09 – 10.3) |  |
|  | No | 5 (0 – 10) |  | 1 |  |
|  | Disease |  | 0.07 |  |  |
|  | Lymphoma | 15 (6 – 24) |  |  |  |
|  | Other | 6 (0 – 11) |  |  |  |
|  | HCT-CI |  | 0.046 |  |  |
|  | ≥ 3 | 15 (6 – 24) |  |  |  |
|  | < 3 | 6 (0 – 10) |  |  |  |
|  | CD3 (x10^8^/kg) |  | 0.07 |  |  |
|  | ≥ 220 | 16 (6 – 25) |  |  |  |
|  | < 220 | 5 (0 – 11) |  |  |  |
|  | GVHD prophylaxis |  |  |  | 0.014 |
|  | PTCy-CsA-MMF | 18 (9 – 27) | 0.002 | 12.5 (1.66 – 93.5) |  |
|  | PTCy-Sir-MMF | 2 (0 – 4) |  | 1 |  |
| **SOS** |  |  |  |  |  |
|  | Disease |  | 0.038 |  |  |
|  | Other | 13 (6 – 20) |  |  |  |
|  | Acute leukaemia | 2 (0 – 6) |  |  |  |
|  | Disease stage |  |  |  |  |
|  | Other | 12 (5 – 19) | 0.067 |  |  |
|  | Early | 2 (0 – 7) |  |  |  |
|  | Donor age (years) |  | 0.001 |  |  |
|  | < 40 | 17 (8 – 27) |  | 1.11 (1.04 – 1.19) |  |
|  | ≥ 40 | 1 (0 – 4) |  | 1 |  |
|  | GVHD prophylaxis |  | 0.08 |  | 0.018 |
|  | PTCy-Sir-MMF | 14 (5 – 22) |  | 10.8 (1.52 – 77) |  |
|  | PTCy-CsA-MMF | 4 (0 – 9) |  | 1 | 0.004 |

Abbreviations, AKI: acute kidney injury; SOS: sinusoidal obstruction syndrome; CBF: cyclophosphamide, busulfan and fludarabine; TBF: thiotepa, busulfan and fludarabine; CumInc: cumulative incidence; CI: confidence interval; D/R: donor/receptor; CBF: PTCy-CsA-MMF: postransplant cyclophosphamide, cyclosporine and mycophenolate; PTCy-Sir-MMF: postransplant cyclophosphamide, sirolimus and mycophenolate; SCT: stem cell transplantation; HCT-CI: Hematopoietic Cell Transplantation-specific Comorbidity Index

* Cumulative incidence at 100 days (SOS) or 180 days (AKI and thrombotic microangiopathy)
